# The chemokine CCL14 is a potential biomarker associated with immune cell infiltration in lung adenocarcinoma

**DOI:** 10.1007/s12672-024-01160-4

**Published:** 2024-07-20

**Authors:** Bai-er Sun, Zai-xin Yuan, Meng-jiao Wang, Li-qin Xu, Jian Feng, Jing-jing Chen

**Affiliations:** 1 Department of Respiratory and Critical Care Medicine, Affiliated Hospital of Nantong University, 20 Xi-Si Road, Nantong, 226001 Jiangsu People’s Republic of China; 2 Nantong University, Nantong, Jiangsu China

**Keywords:** Lung adenocarcinoma, C–C motif chemokine ligand 14, Tumor-infiltrating immune cells, Bioinformatics, Cell experiment

## Abstract

**Background:**

Chemokine ligand 14, which has a C–C motif (CCL14), mediates the immunological milieu around tumors. However, its role in the progression of lung adenocarcinoma (LUAD) is still unknown. Our objectives were to study the association between CCL14 and tumor-infiltrating immune cells (TIICs) as well as the predictive significance of CCL14 in LUAD.

**Methods:**

The expression of CCL14 in LUAD was examined by using the Oncomine, The Cancer Genome Atlas (TCGA), The University of Alabama at Birmingham CANcer data analysis Portal (UALCAN), and Human Protein Atlas databases. To determine the prognostic significance of CCL14 in LUAD, researchers used the Kaplan‒Meier plotter and Gene Expression Profiling Interactive Analysis (GEPIA, version 2). We utilized TIMER and GEPIA2 to investigate the connection between CCL14 and TIICs. Gene set enrichment analysis (GSEA) was used to test for functional enrichment of genes. We used RT‒qPCR to measure CCL14 expression and Cell Counting Kit-8, Transwell, and wound healing assays to investigate the biological role of CCL14.

**Results:**

The prognosis of patients with LUAD was worse when CCL14 expression was low. Statistical analysis revealed that CCL14 mRNA expression was significantly greater in lung epithelial cells than in LUAD cell lines in vitro. Enhancing CCL14 expression reduced cell migration, invasion, and proliferation. The results of the immune infiltration research showed that CCL14 and TIICs were positively correlated. Different immune infiltration patterns associated with CCL14 were also shown by TIIC markers. According to GSEA, histone deacetylases, G2/M checkpoints, and Notch signaling pathways were associated with low CCL14 expression.

**Conclusions:**

CCL14 is anticipated to emerge as a prognostic marker and therapeutic target for LUAD due to its role in regulating TIICs, suggesting that it may be an antioncogene.

## Introduction

Responsible for an estimated 1.8 million fatalities (18% of cancer cases) annually, lung cancer is still one of the most common cancers to be diagnosed and the world’s leading cause of cancer-related deaths overall [1]. Forty percent of lung cancer cases are lung adenocarcinoma (LUAD), the most common histologic subtype of lung cancer [2]. Advances in precision medicine have made immunotherapy and targeted therapy more important in advanced LUAD. However, LUAD has a bleak outlook, and the 5-year survival rate varies with disease and treatment [1]. Therefore, discovering new immunological pathways and therapeutic targets is an urgent matter.

Scientists believe that tumor cells, stromal fibroblasts, and immune cells all interact in complex ways throughout the development of lung cancer [3]. Tumor-infiltrating immune cells (TIICs) are important for either stimulating or suppressing the growth of tumors [4]. In the tumor microenvironment, chemokines are essential for immune cell recruitment to the tumor microenvironment. Chemokines play multiple and complex roles in the tumor microenvironment [5]. They regulate the chemotaxis and recruitment of various immune cells, directly or indirectly influence tumor cell growth, invasion, and metastasis, and modulate tumor angiogenesis [6]. Certain chemokines (such as CXCL9 and CXCL10) can promote anti-tumor immune responses, while others (like CCL2 and CXCL12) may facilitate tumor progression [7]. Tumor cells themselves can also regulate chemokine expression through mechanisms such as epigenetic modification. This complex network of interactions makes chemokines important subjects for understanding the tumor immune microenvironment and developing novel immunotherapy strategies. However, it also suggests that targeting the chemokine network requires careful consideration of its multifaceted nature and potential side effects [8].

Chemokines can be broadly categorized into four groups: CXC, CC, C, and CX3C [8]. CC-type chemokine C–C motif chemokine ligand 14 (CCL14) is constitutively expressed in multiple organs [9]. Gu et al. reported strong negative correlations between CCL14 expression and the infiltration of B cells, CD4 + and CD8 + T cells, macrophages, neutrophils, and dendritic cells (DCs) in hepatocellular carcinoma (HCC). Previous studies have demonstrated the significance of CCL14 in various cancers, including HCC [9], ovarian cancer, and thyroid cancer [10, 11]. In these malignancies, CCL14 expression was generally downregulated in tumor tissues and associated with better survival outcomes and less advanced disease. Notably, CCL14 was found to correlate with immune cell infiltration and potentially modulate the tumor immune microenvironment. These findings provide a valuable comparative framework for investigating the role of CCL14 in LUAD, particularly its expression patterns, prognostic value, and potential immune regulatory functions.

Despite advancements in precision medicine for LUAD treatment, the need for new immunological targets remains critical. CCL14, a chemokine known to modulate tumor immunity in various cancers, has not been thoroughly investigated in LUAD. Given its potential to influence immune cell infiltration and patient outcomes, we conducted a comprehensive analysis of CCL14 in LUAD. We investigated CCL14 expression and its association with patient prognosis using databases including Oncomine, UALCAN, and GEPIA, with further analysis using the Kaplan- Meier plotter. Additionally, we performed in vitro studies of CCL14 expression and function in LUAD cell lines. We examined the connection between CCL14, and tumor-infiltrating immune cells using TIMER, conducted a protein–protein interaction network analysis, and used gene set enrichment analysis to determine CCL14’s biological signaling pathways. This comprehensive approach aims to provide new insights into CCL14’s role in LUAD and its potential as a diagnostic, prognostic, and therapeutic target, contributing to the development of novel strategies for this challenging malignancy.

## Materials and methods

### Data acquisition

Clinical data and CCL14 gene expression profiles from datasets from patients with LUAD were obtained from TCGA, which is available to the general public. Patients who did not meet the exclusion criteria were those who lacked sufficient or missing information on clinical characteristics and overall survival. Based on the expression of CCL14 in additional studies, the above data were divided into two groups.

### CCL14 gene expression analysis

In a number of malignancies, including LUAD, as well as in matched normal tissues, the levels of CCL14 mRNA expression were investigated using the Oncomine Platform (https://www.oncomine.org/resource/login.html). In this study, we selected a 1.5-fold change (*P* value = 1E−3). In addition, the “Diff exp module” of the TIMER database (https://cistrome.shinyapps.io/timer/), the “CPTAC analysis module” of the UALCAN database (http://ualcan.path.uab. edu/analysis.html), and the Human Protein Atlas (HPA) database (http://www.proteinatlas.org) were used to further investigate the protein levels of CCL14 in both normal lung tissues and LUAD tissues. We used a receiver operating characteristic (ROC) curve to assess how well the expression level of CCL14 discriminates between LUAD and normal tissues.

### Survival analysis

We examined the predictive significance of CCL14 mRNA expression in LUAD and LUSC using GEPIA2 (http://gepia2.cancer-pku.cn/, version 2) and the Kaplan‒Meier Plotter (http://kmplot.com/analysis/). We chose “median” as the group cutoff and “LUAD” as the cancer name in the “Survival” module of GEPIA2. We used the hazard ratio (HR) and the log rank p value to determine the association between CCL14 expression and lung cancer prognosis. The Kaplan–Meier plotter was used to automatically plot the data.

### Clinical significance analysis of CCL14 expression in LUAD

The clinical significance of CCL14 expression in LUAD was analyzed using public data to examine its differential expression across various clinical variable groups. The analysis was performed using R version 3.5.3, with the ggplot2 (version 3.3.6), stats (version 3.5.3), and car (version 3.1–0) packages. The Kruskal–Wallis test was employed as the overall test, followed by Dunn's test for multiple hypothesis testing. The clinical variables investigated included pathological TNM stage, pathological stage (I-IV), primary therapy outcome, gender, race, age, residual tumor, anatomic neoplasm subdivision, location, smoker status, and number of pack-years smoked.

### Cell lines and cell culture

We purchased the BEAS-2B lung epithelial cell line, the NCI-H1299 human non-small cell lung cancer cell line, and the PC9 human non-small cell lung cancer cell line from ScienCell (Carlsbad, CA, USA). Data for cell typing and mycoplasma tests were provided by the institution. The cells were cultured in RPMI-1640 medium (Gibco, USA) supplemented with 10% fetal bovine serum (FBS (Gibco, USA)), 2 mM L-glutamine, and 100 U/ml penicillin/streptomycin (Gibco, USA). The cells were incubated at 37 °C in a humidified environment with 5% CO_2_. A regular mycoplasma test was performed on cell cultures as a standard quality assurance measure.

### Reverse transcription–quantitative PCR (RT‒qPCR)

RNA was isolated from cells using TRIzol reagent (Invitrogen, USA) and then converted into cDNA using a RevertAid First Strand cDNA Synthesis Kit (Thermo Fisher Scientific, USA) following the manufacturers’ guidelines. The RT‒PCR primers were purchased from Sangon Biotech (Shanghai, China): CCL14 forward: 5′-TGCTGCTTCACCTACACTACCTAC-3′, CCL14 reverse: 5′-CACTCAGTTCTCCTTCATGTCCTTG-3′; and GAPDH forward: 5′- CAGGAGGCATTGCTGATGAT-3′, GAPDH reverse: 5′-GAAGGCTGGGGCTCATTT-3′. RT‒qPCR was conducted on an ABI 7500 FAST Real-Time PCR System from Applied Biosystems (Carlsbad, CA, USA) using SYBR Green Master Mix from Vazyme (Nanjing, China). The mRNA levels were measured using the 2–∆∆Ct technique following normalization to GAPDH mRNA.

### Transfection of the CCL14 overexpression plasmid

Vigene Biosciences (WZ Biosciences Inc., Jinan, China) supplied the vehicle vector and plasmids for miR-CCL14 overexpression. We transported the sequences into cells by inserting them into the PGPH1/GFP/puro vector. We used Lipofectamine^™^ 3000 (Invitrogen, USA) to transfect the cells. Transfection efficiency was evaluated by measuring CCL14 mRNA levels in LUAD cells before and after transfection using RT‒qPCR.

### Cell growth and viability assay

A Cell Counting Kit-8 (CCK-8) from Beyotime Institute of Biotechnology in Shanghai, China, was used to inspect the proliferation and health of cells in accordance with the instructions provided by the manufacturer. The cells were prepared as a suspension by digestion, centrifugation, and transformation. At a density of 5 × 10^3^ cells per well, 96-well plates were seeded with cells after counting with a cell counting board. After 6–8 h of growth, the cells were allowed to adhere to the plates in a humidified 5% CO_2_ incubator at 37 °C. The wells were incubated for 2 h after the addition of 10 μl of CCK-8 solution at each time point, ranging from 24 to 96 h. The absorbance was measured at 450 nm with a Synergy HT microplate spectrophotometer from Biotech, USA.

### Cell migration and invasion assays

The invasion and migration of cells were evaluated with the use of 24-well plates and Transwell chambers (353097, Falcon, USA). The migration test was conducted by adding 600 µl of 20% FBS-containing medium to the bottom chamber. Next, the upper chamber was filled with 100 μl of cell suspension, which had a cell density of 5 × 10^4^ cells/chamber. The containers were placed in a cell incubator for one day. After the incubation was complete, the upper chamber medium was removed, and a cotton swab was used to wipe down the cells and any liquid that may have accumulated on the surface. After invading cells were stained with 0.3% crystal violet (Sigma-Aldrich, USA), they were fixed with 4% paraformaldehyde. To assess invasion, a light microscope (Olympus, Japan) was used to count the cells after five randomly selected visual fields (magnification × 200) were examined for each implant. Matrigel from BD Biosciences (San Jose, CA, USA) was prepared according to the manufacturer’s instructions and used in the invasion assay. Three separate experiments were carried out.

### Wound-healing assay

To evaluate cell migration, a wound healing assay was conducted. The cells were seeded in 6-well plates. A vertical scratch was made in each 6-well plate, and the plates were rinsed with PBS to remove any detached cells once the cell confluence reached 95%. Five different fields of cells were photographed again after 36 h of incubation. The migration rate (MR) was assessed by measuring the wound width and then dividing it by the initial measurement (D0) of the wound width at 0 h and the second measurement (D1) of the wound width at 36 h.

### Tumor-infiltrating immune cells

To study the relationship between CCL14 expression and the amount of immune infiltrates in LUAD, the TIMER database was used as a total resource. Through the “Gene” and “Correlation” modules, we identified immunological infiltrates containing B cells, CD4 + T cells, CD8 + T cells, neutrophils, macrophages, and dendritic cells. Using GEPIA2’s “Correlation” module, we double-checked the association between CCL14 expression and TIICs. The relationships between CCL14 expression and TIIC markers were examined using Spearman’s correlation analysis. According to the following r values, the correlations were classified as very weak (0.00–0.19), weak (0.20–0.39), moderate (0.40–0.59), strong (0.60–0.79), or very strong (0.80–1.0). We next compared the levels of tumor infiltration with various changes in the somatic copy number of CCL14 using the “SCNA” module.

### Differential expression, functional enrichment, and pathway analyses

For the differential gene expression analysis of CCL14 in TCGA-LUAD, the DESeq2 pipeline was employed. Samples were divided into high expression (50–100%) and low expression (0–50%) groups based on CCL14 gene expression levels, with the low expression group serving as the reference. Differential analysis was then performed on the raw counts matrix using both DESeq2 (version 1.36.0) and edgeR (version 3.38.2) packages in R (version 3.5.3). Differentially expressed genes were identified using the criteria of |logFC|> 1 and adjusted p-value < 0.05, resulting in 1035 genes.

Functional enrichment analysis was conducted on the differentially expressed genes using the clusterProfiler R package, focusing on Gene Ontology (GO) and Kyoto Encyclopedia of Genes and Genomes (KEGG) databases. To facilitate analysis, gene IDs were converted to corresponding Entrez IDs using the org.Hs.eg.db package, achieving a conversion rate of 98.1%. The enrichment analysis encompassed Biological Process (BP), Cellular Component (CC), Molecular Function (MF), and KEGG pathways, with significance typically determined by an adjusted p value < 0.05. The top 20 terms for each category (BP, CC, MF, and KEGG) were selected based on ascending adjusted p-values and presented in the results. Pairwise similarities between enriched terms were calculated using Jaccard's similarity index (JC). Hierarchical clustering was performed on these similarity results using hclust, and the clustering outcomes were visualized using the ggplot2 package (version 3.3.6).

Gene Set Enrichment Analysis (GSEA) was performed to explore the biological signaling pathways involved in the low- and high-expression groups relative to the median level of CCL14 expression. The analysis utilized 1000 gene set permutations to detect pathways with substantial differences between the groups. Enhanced pathways were ranked using the normalized enrichment score (NES) and the nominal P value. Significant gene sets were defined as those with a false discovery rate (FDR) lower than 0.05.

### Interactions among chemokines

Using the STRING database (https://string-db.org/), we conducted an analysis of the protein‒protein interaction network involving differentially expressed chemokines. To begin the analysis, we set the minimum needed interaction scores at “medium confidence (0.400)” and “high confidence (0.700)”.

### Statistical analysis

Statistical analyses were performed using R version 3.5.3 (www.r-project.org) and GraphPad Prism 8.0 (GraphPad Software, San Diego, CA, USA). Experimental results are presented as the mean ± SEM (Standard Error of the mean), derived from three independent experiments, each performed in duplicate. For comparisons between two groups, two-tailed Student's t-tests were used. One-way ANOVA followed by Tukey’s post-hoc test was applied for multiple group comparisons. In bioinformatics analyses involving multiple comparisons, p-values were adjusted using the Benjamini–Hochberg method to control the false discovery rate. All statistical tests were two-sided, and p < 0.05 was considered statistically significant. Both unadjusted (p) and adjusted p-values (padj) are reported where applicable. Significance levels are denoted as follows: *p/padj < 0.05, **p/padj < 0.01, ***p/padj < 0.001.

## Results

### Evaluation of CCL14 expression in various normal and cancerous tissues

**Fig. 1:**
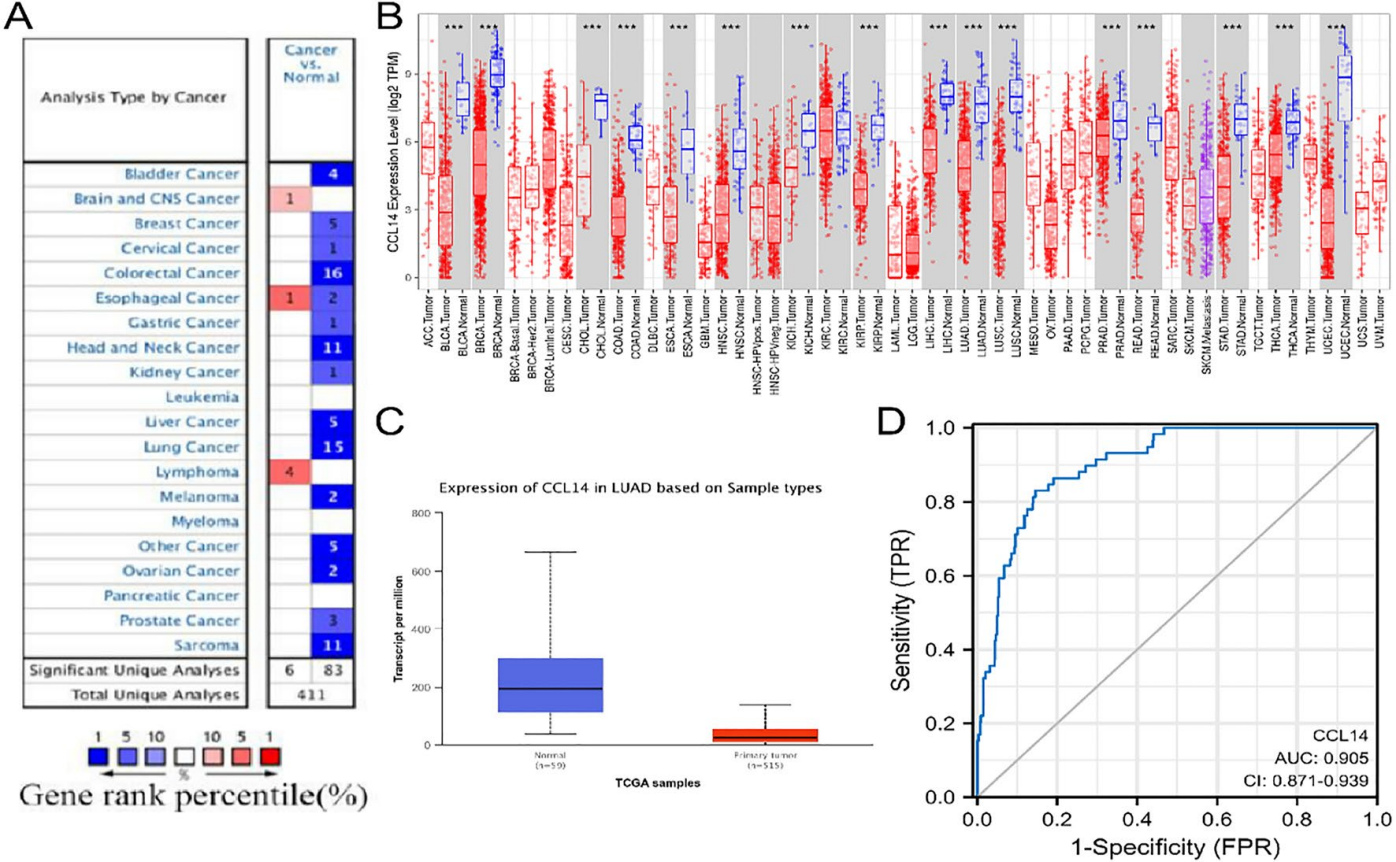
CCL14 expression in human tumor tissues (**A** and **B**) High or low expression of CCL14 in different human cancer tissues compared with normal tissues according to the Oncomine database (**A**) and TIMER database (**B**). **C** CCL14 expression analysis in LUAD and normal tissues using the UALCAN database. **D** ROC curve for the effectiveness of CCL14 levels in distinguishing LUAD tissues from normal tissues

We used the Oncomine database to evaluate CCL14 expression in different types of cancer tissues and healthy tissues. Our results showed that CCL14 expression was greater in brain tumor, esophageal cancer, and lymphoma tissues than in normal tissue samples. Figure 1A shows that compared to normal tissue controls, patients with bladder, breast, lung, stomach, liver, or colorectal cancer exhibited reduced CCL14 expression. When looking for differences in CCL14 expression between certain tumor types, we used the TIMER and UALCAN databases. CCL14 expression levels were significantly lower in patients with eleven malignancies, including LUAD, than in healthy controls. The differences in CCL14 expression between UALCAN samples of normal adjacent tissue and LUAD tissues are shown in Fig. 1C. Compared to cancerous lung tissues, normal lung tissues showed greater CCL14 expression, as revealed by HPA.

Using ROC curves, we performed an in-depth evaluation of the ability of CCL14 expression levels to distinguish LUAD tissues from normal tissues. Figure 1D shows the results, which revealed an area under the curve (AUC) of 0.905 (95% CI = 0.871–0.939), a sensitivity of 0.864, a specificity of 0.837, and statistical significance (P < 0.05). These findings raise the possibility that CCL14 might be a biomarker for differentiating LUAD tissue from healthy tissue.

**Fig. 2:**
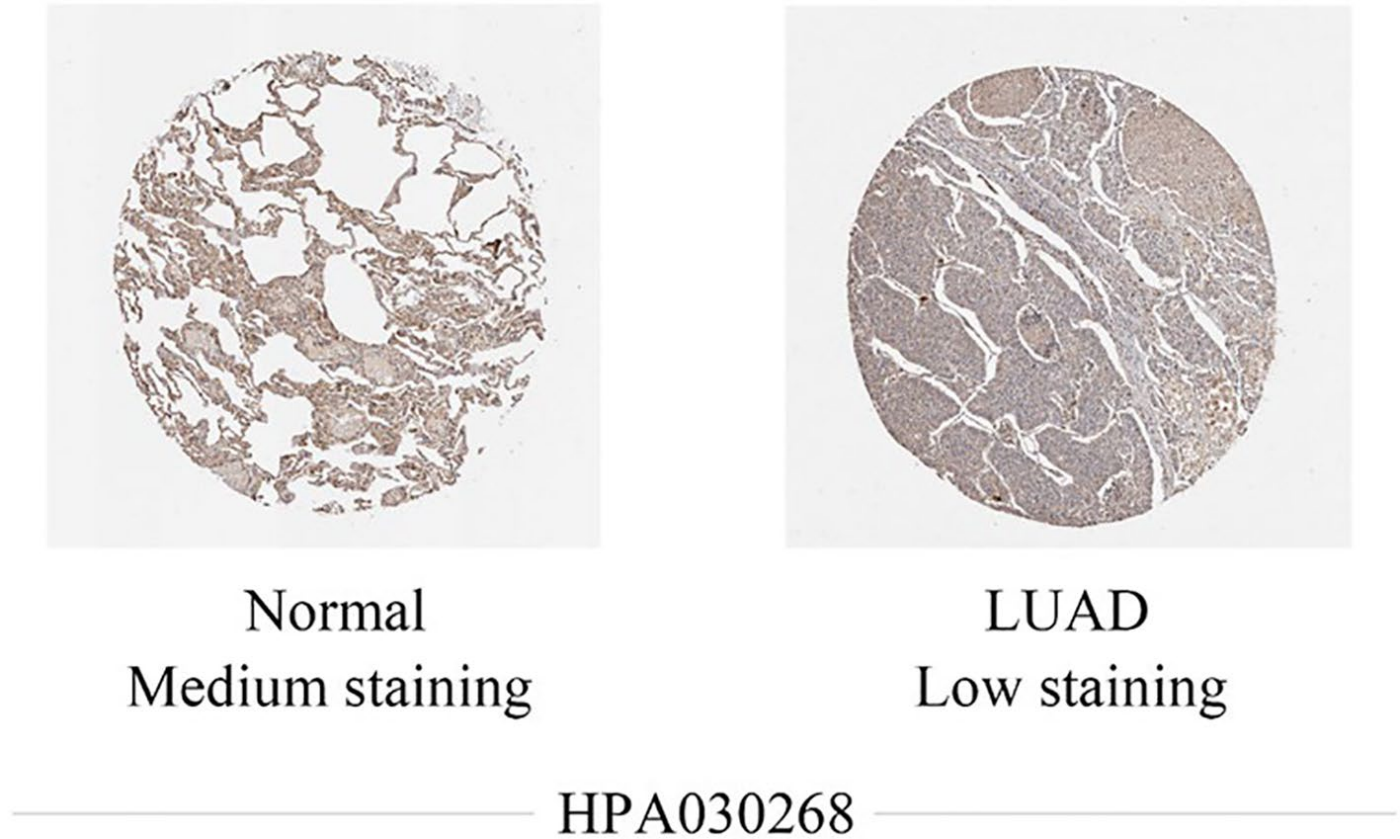
Representative images of CCL14 protein immunohistochemical staining of normal lung tissues and LUAD tissues in the HPA database

Figure 2 presents representative immunohistochemical staining images of CCL14 protein expression in normal lung tissues and lung adenocarcinoma (LUAD) tissues from the Human Protein Atlas (HPA) database. Normal lung tissues exhibited medium CCL14 protein staining. In contrast, LUAD tissues showed low CCL14 protein staining in tumor cells. This visible difference in CCL14 protein levels between normal and LUAD tissues aligns with the mRNA expression data presented in Fig. 1, further supporting the downregulation of CCL14 in LUAD and suggesting its potential role in lung adenocarcinoma development or progression.

### Correlation between CCL14 expression and the clinicopathological characteristics of patients with LUAD

Using the publicly available TCGA database, we collected clinical data and data on CCL14 gene expression patterns in patients with LUAD. A comprehensive set of 538 cases was obtained from TCGA, with the exclusion of those that lacked prognostic information and clinical characteristics. We ultimately enrolled 535 patients who met the inclusion criteria and performed logistic regression analysis to investigate the correlations between CCL14 expression and the clinicopathological characteristics of patients with LUAD. CCL14 expression was categorized into two groups based on its median value: high expression and low expression. The detailed results of the correlation analysis between CCL14 expression and clinicopathological characteristics are presented in Table 1. Notably, increased CCL14 expression was significantly correlated with tumor status (T2, Stage III and Stage IV vs. Stage I, OR = 0.681, 95% CI = 0.484–0.958, P = 0.028), sex (male vs. female, OR = 0.681, 95% CI = 0.484–0.958, P = 0.028), and earlier pathological stage (T2, T3 and T4 vs. T1; OR = 0.459, 95% CI = 0.315–0.663, P < 0.001). In contrast, the expression of CCL14 did not significantly differ according to race, age, lymph node metastasis status, or distant metastasis status.

**Table 1:** Correlation analysis between CCL14 expression and clinicopathological characteristics of patients with LUAD in TCGA

Characteristic	Total (N)	Odds ratio (OR)	*P* value
T stage (T2&T3&T4 vs. T1)	532	0.459 (0.315–0.663)	< 0.001^*^
N stage (N1&N2&N3 vs. N0)	519	0.765 (0.529–1.103)	0.152
M stage (M1 vs. M0)	386	0.591 (0.245–1.347)	0.221
Pathologic stage (stage II & stage III & stage IV vs. stage I)	527	0.632 (0.446–0.893)	0.009^*^
Sex (male vs. female)	535	0.681 (0.484–0.958)	0.028^*^
Race (Asian & Black or African-American vs. White)	468	0.875 (0.511–1.495)	0.625
Age (> 65 vs. < = 65)	516	1.302 (0.922–1.842)	0.135

*CCL14* C–C motif chemokine ligand 14, *LUAD* lung adenocarcinoma

^*^P < 0.05

### Prognostic value of CCL14 expression in patients with LUAD

The research team investigated the relationship between CCL14 expression and prognosis in patients with LUAD utilizing multivariate and univariate Cox regression analyses. The findings from the univariate Cox regression analysis, which are presented in Table 2, indicated that the following factors influenced the study outcomes: tumor status (T2 & T3 & T4 vs. T1, HR = 1.728, 95% CI = 1.229–2.431, *P* = 0.002), distant metastasis (2.136, 95% CI = 1.248–3.653, *P* = 0.006), pathologic stage (stage II & stage III & stage IV vs. stage I, HR = 2.933, 95% CI = 2.173–3.958, *P* < 0.001), and CCL14 expression level (high-expression group). Tumor status (T2 & T3 & T4 vs. T1, HR = 1.658, 95% CI = 1.055–2.605, *P* = 0.028), lymph node metastasis status (HR = 1.833, 95% CI = 1.027–3.273, *P* = 0.040), and CCL14 expression level (high-expression group vs. low-expression group, HR = 0.896, 95% CI = 0.814–0.985, *P* = 0.023) were identified as independent factors for OS by multivariate Cox regression analysis.

**Table 2:** Correlation of CCL14 expression and overall survival in patients with LUAD with different clinicopathological factors according to univariate (A) and multivariate (B) Cox regression analyses

Clinicopathologic variable	HR (95% CI)	*P* value
A		
Age	1.223 (0.916–1.635)	0.172
Gender	1.070 (0.803–1.426)	0.642
T (tumor status)	1.728 (1.229–2.431)	0.002^*^
N (lymph node)	2.601 (1.944–3.480)	< 0.001^*^
M (distant metastasis)	2.136 (1.248–3.653)	0.006^*^
Pathologic Stage	2.933 (2.173–3.958)	< 0.001^*^
CCL14	0.913 (0.847–0.984)	0.017^*^
B		
T (tumor status)	1.658 (1.055–2.605)	0.028^*^
N (lymph node)	1.833 (1.027–3.273)	0.040^*^
CCL14	0.896 (0.814–0.985)	0.023^*^

A. Univariate Cox regression analysis. B. Multivariate Cox regression analysis

*CCL14* C–C motif chemokine ligand 14, *LUAD* lung adenocarcinoma

^*^P < 0.05

**Fig. 3:**
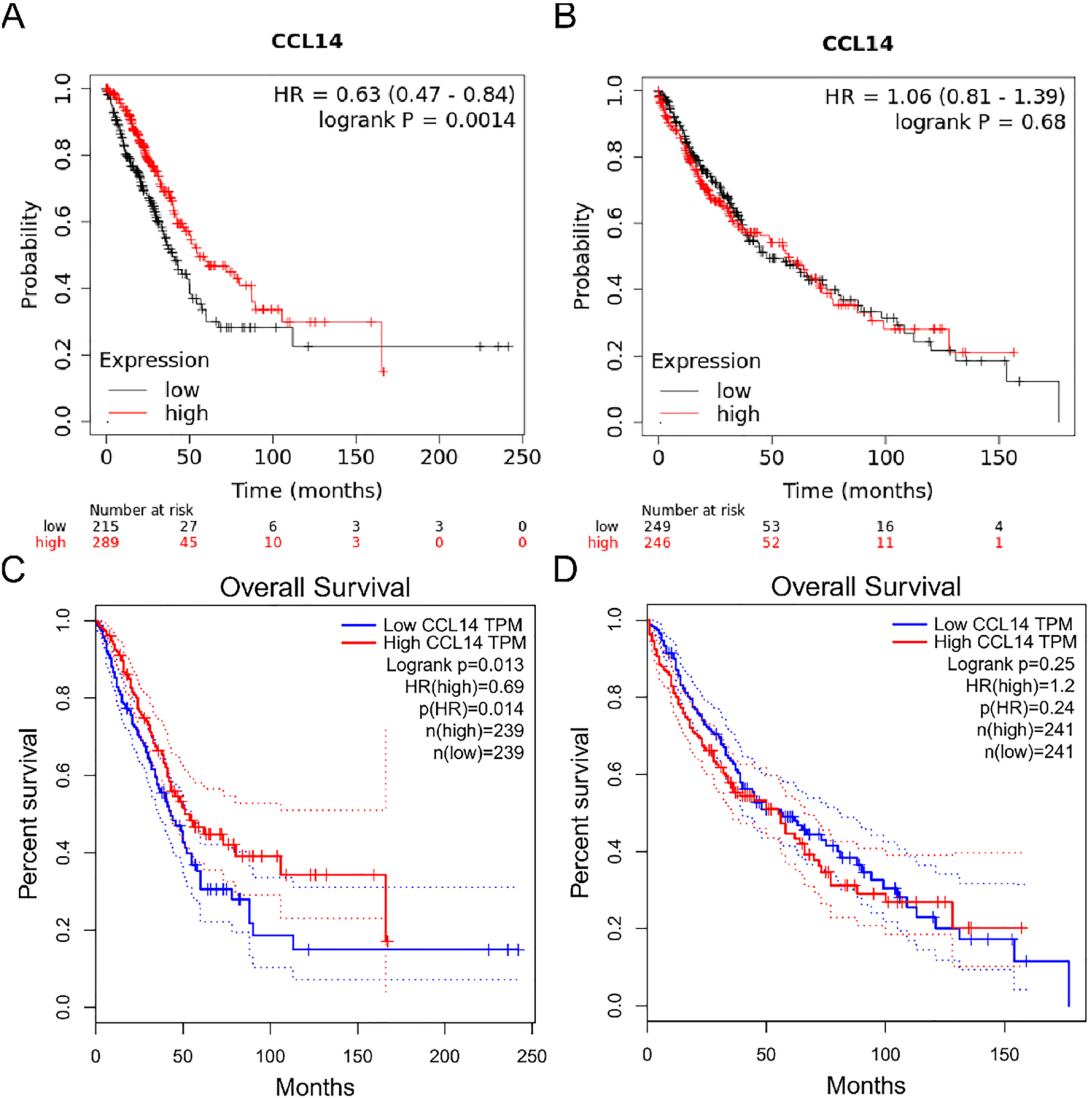
Kaplan–Meier survival curve analysis of the prognostic significance of high and low CCL14 expression in lung cancers using the Kaplan‒Meier plotter database and the GEPIA database. **A** and **B** Kaplan‒Meier survival curve analysis of the prognostic significance of high and low expression of CCL14 in LUAD (**A**) and LUSC (**B**) using the Kaplan‒Meier plotter database. **C** and **D** Kaplan‒Meier survival curve analysis of the prognostic significance of high and low expression of CCL14 in LUAD **(C)** and LUSC (**D**) using the GEPIA database

The survival graphs for the respective analyses were subsequently generated utilizing the Kaplan‒Meier plotter and the GEPIA2 database. A poor prognosis was associated with low CCL14 expression in patients with LUAD according to data from both databases (Fig. 3A and C, P < 0.05). However, no statistically significant correlation was observed between CCL14 expression and the prognosis of patients diagnosed with LUSC (Fig. 3B and D, P > 0.05).

### Association of CCL14 expression with clinical characteristics in LUAD patients

**Fig. 4:**
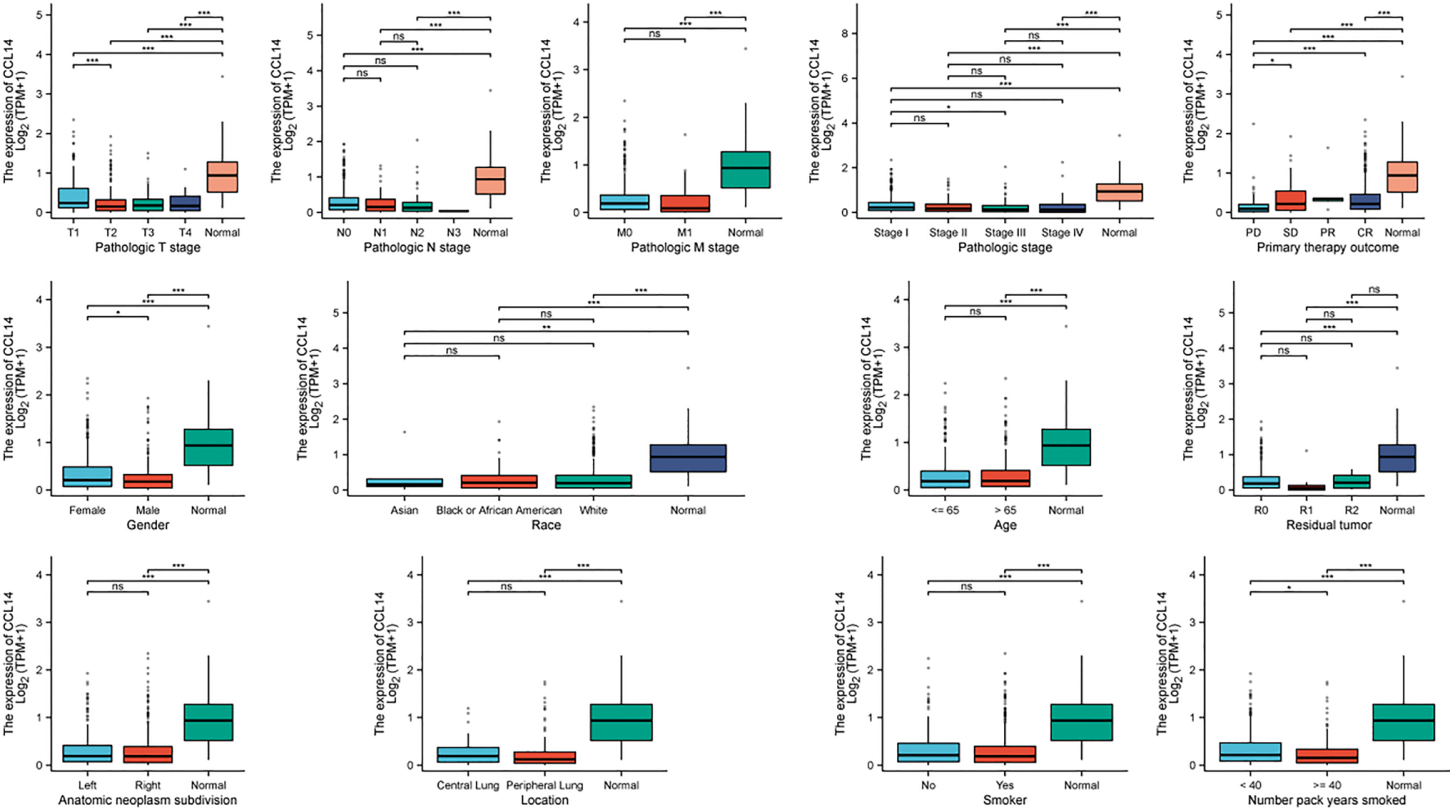
Association of CCL14 expression with clinical characteristics in LUAD patients. Box plots show CCL14 expression levels [Log2(TPM + 1)] across various clinical variables: pathologic T stage, pathologic N stage, pathologic M stage, pathologic stage, primary therapy outcome, gender, race, age, residual tumor, anatomic neoplasm subdivision, tumor location, number of pack years smoked, and smoker status

The analysis of CCL14 expression across various clinical characteristics in LUAD patients revealed several significant findings (Fig. 4). CCL14 expression was significantly lower in pathological T2 stage compared to T1 stage, while no significant differences were observed across pathological N stages. Similarly, no significant difference was found between M0 and M1 stages. Patients with pathological stage III showed significantly lower CCL14 expression than those in stage I. Regarding primary therapy outcomes, both SD (stable disease) and CR (complete response) groups exhibited significantly higher CCL14 expression compared to the PD (progressive disease) group. Female patients had significantly lower CCL14 expression than male patients. No significant differences were observed among different racial groups (Asian, Black or African American, White) or between patients above and below 65 years of age. CCL14 expression did not differ significantly among R0, R1, and R3 residual tumor groups, nor between left and right lung tumors. Central and peripheral lung tumors showed no significant difference in CCL14 expression. While smoking status did not affect CCL14 expression, patients who smoked 40 or more pack-years had significantly lower CCL14 expression compared to those who smoked less than 40 pack-years.

### Expression of CCL14 in LUAD cell lines

**Fig. 5:**
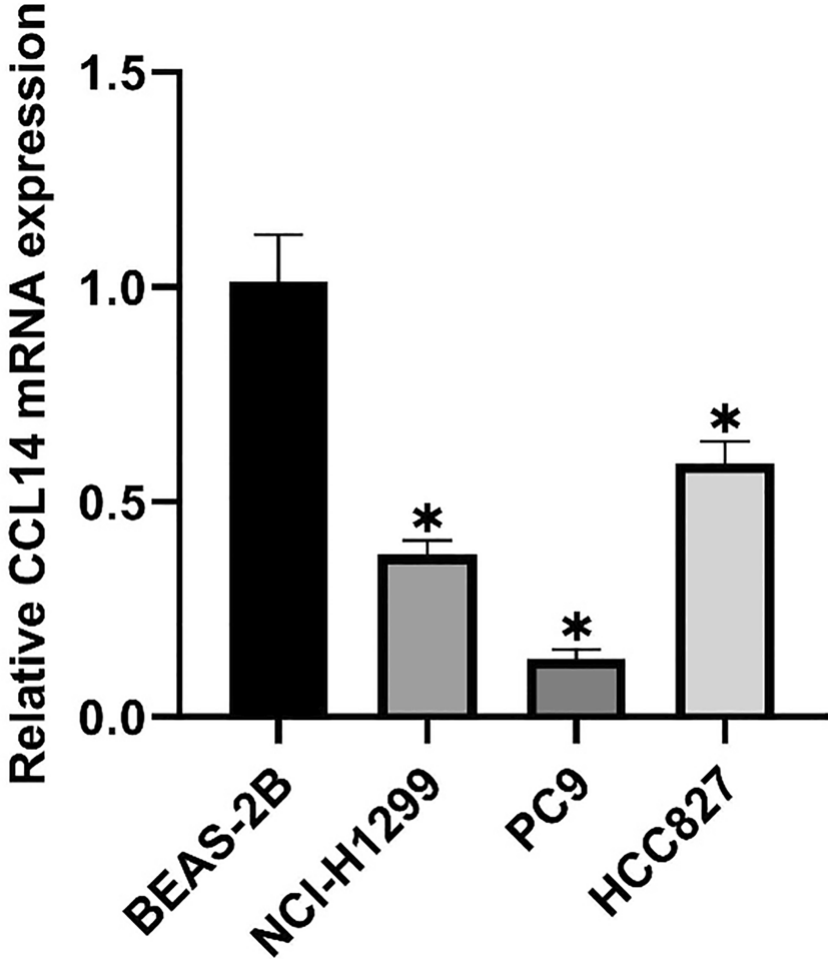
RT‒qPCR analysis of CCL14 mRNA expression in lung epithelial cells (BEAS-2B) and LUAD cell lines (NCI-H1299, PC9, HCC827). n = 6, *P < 0.05

By using qPCR, we confirmed that CCL14 was expressed in both normal samples and LUAD cell lines (NCI-H1299 and PC9 cells). Figure 5 shows that compared with the expression levels in LUAD cell lines (NCI-H1299, PC9, and HCC827 cells, all P < 0.05), the mRNA expression of CCL14 in lung epithelial cells (BEAS-2B) was significantly greater. Specifically, NCI-H1299 and PC9 cells, whose expression levels were quite low, were chosen for the following rounds of cell studies.

### Transfection of the CCL14 overexpression plasmid

**Fig. 6:**
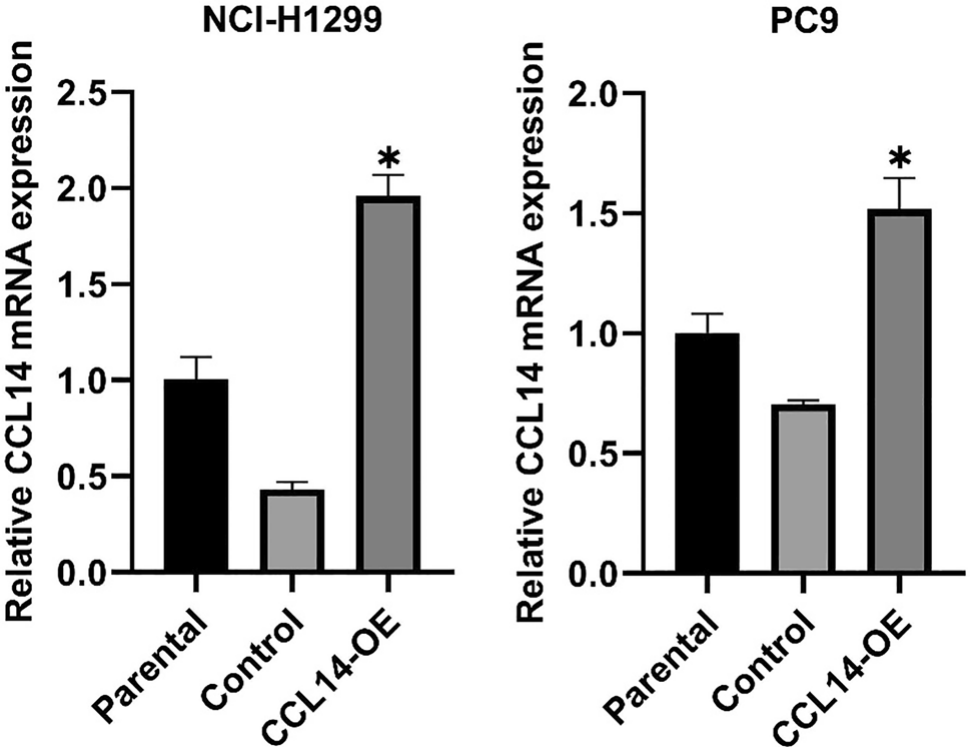
RT‒qPCR analysis of CCL14 mRNA expression in NCI-H1299 and PC9 cells after transfection with the miR-CCL14 overexpression vector plasmid. n = 6, *P < 0.05

We transfected LUAD cell lines with a miR-CCL14 overexpression vector plasmid to enhance CCL14 expression. RT-qPCR was performed to confirm the success of the transfection. Figure 6 shows that subsequent transfection with the miR-CCL14 overexpression vector plasmid resulted in significantly greater CCL14 mRNA expression levels in NCI-H1299 and PC9 cells (both *P* < 0.05).

### Overexpression of CCL14 inhibits the proliferation of LUAD cell lines

**Fig. 7:**
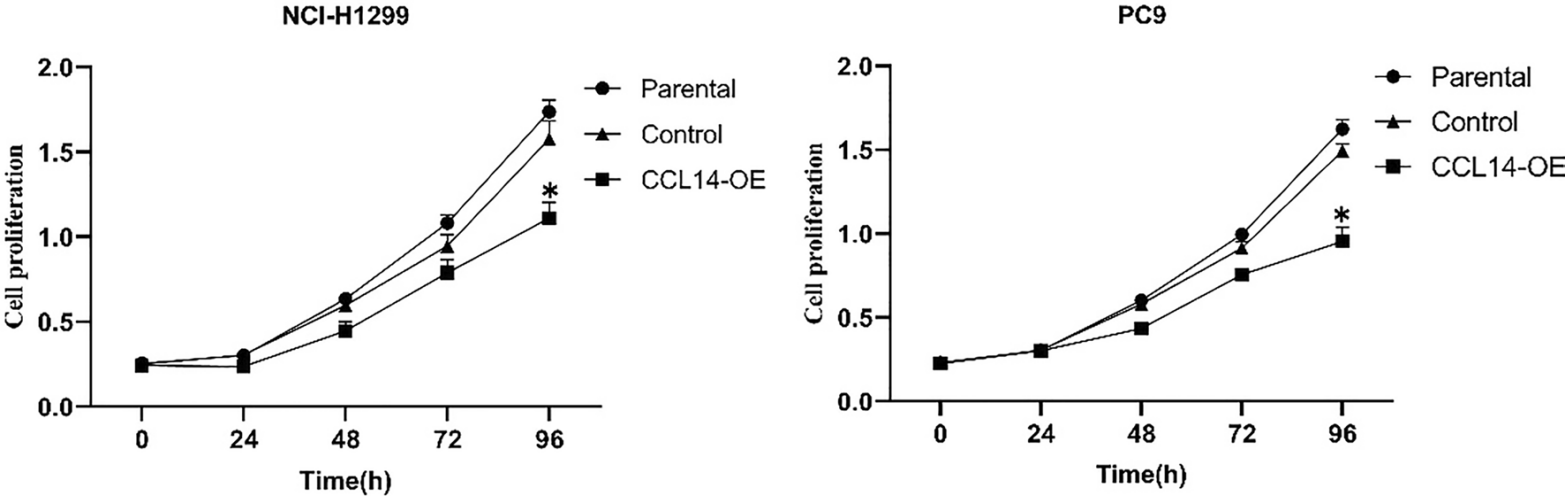
Cell Counting Kit-8 proliferation assays of parental, control-expressing, and CCL14-overexpressing NCI-H1299 and PC9 cells over 96 h. n = 6, *P < 0.05

Excessive proliferation of tumor cells is a characteristic that is commonly observed in cancer. Using the CCK-8 assay, the involvement of CCL14 in the proliferation of LUAD cells was investigated in vitro. Compared with those of the control group, plasmid transfection with the miR-CCL14 overexpression vector significantly reduced the growth of LUAD cell lines (PC9 and NCI-H1299 cells) from 24 to 96 h (P < 0.05) (Fig. 7).

### Overexpression of CCL14 inhibits the migration and invasion of LUAD cell lines

**Fig. 8:**
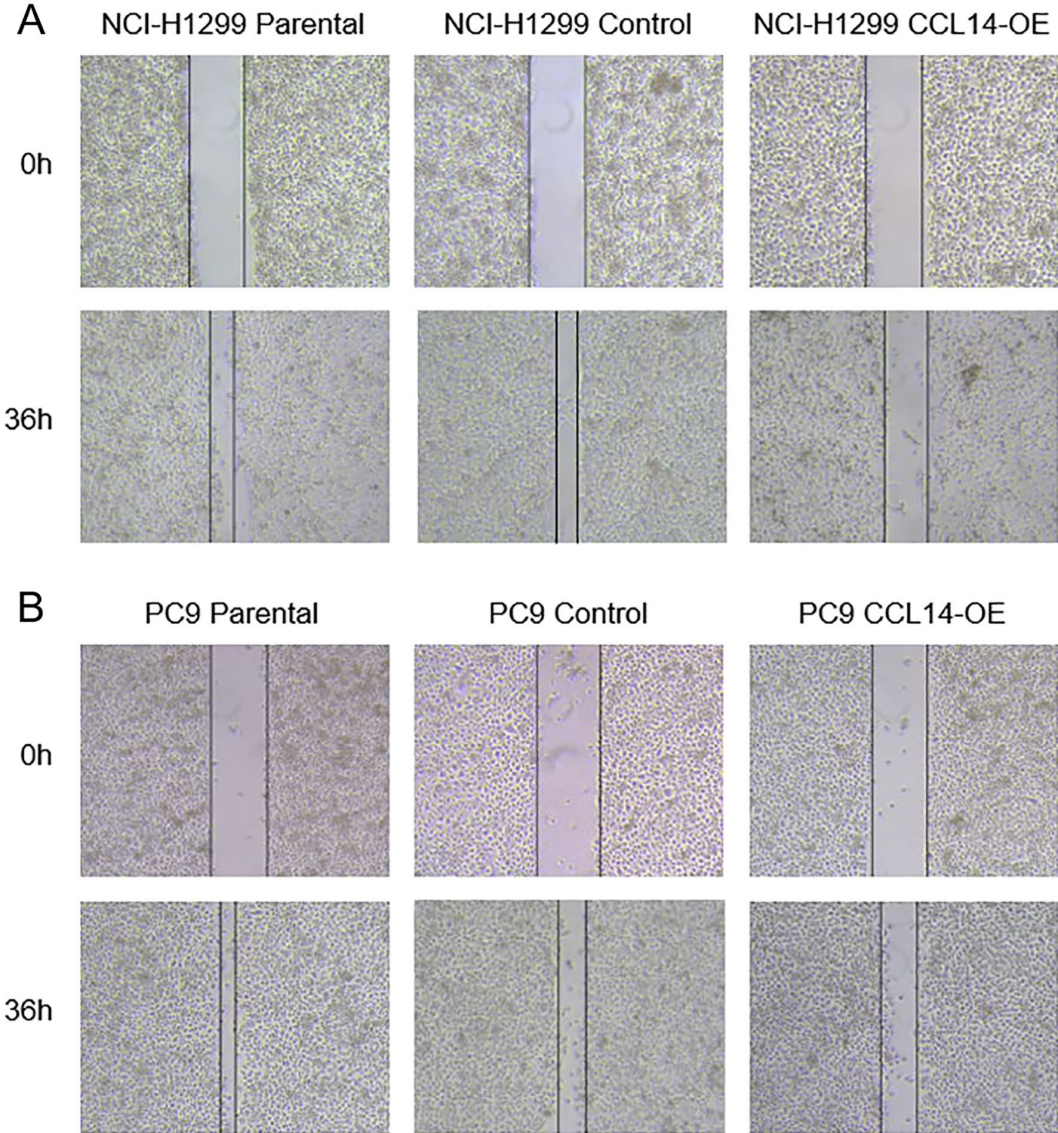
Wound-healing assays of parental, control-expressing, and CCL14-overexpressing (**A**) NCI-H1299 and (**B**) PC9 cells. Each wound width was measured at 0 h and 36 h

**Fig. 9:**
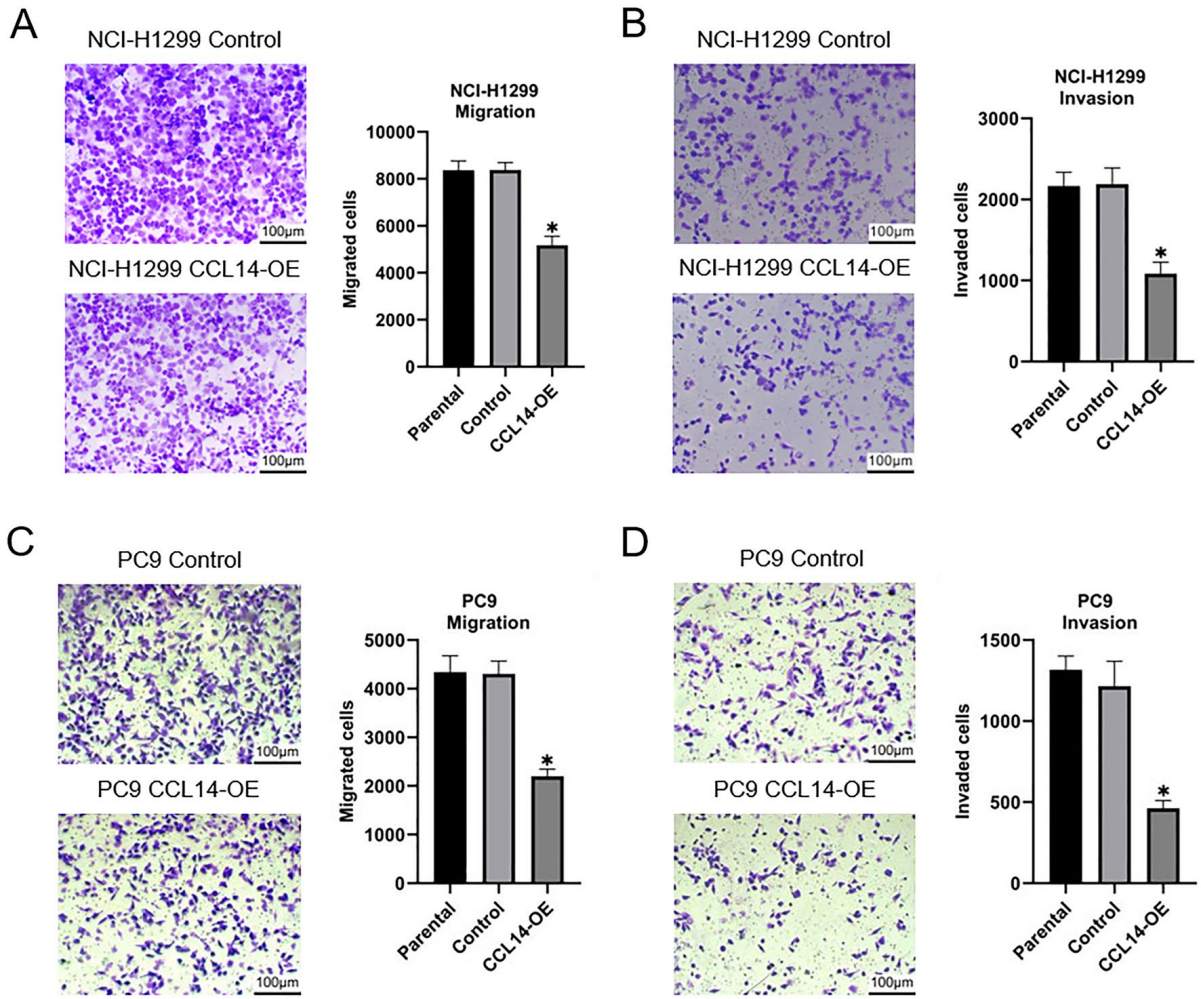
Migration (**A** and **C**) and invasion (**B** and **D**) assays of parental, control-expressing, and CCL14-overexpressing (**A** and **B**) NCI-H1299 and (**C** and **D**) PC9 cells. Graphs were made based on the numbers of migrated and invaded cells. n = 6, * P < 0.05

In advanced stages of disease, tumors may exhibit an elevated propensity for metastasis or postoperative recurrence. Therefore, wound healing and Transwell assays were performed to examine the potential impact of CCL14 on the migration and invasion of LUAD cells. The wound healing assay showed that the group overexpressing CCL14 had greatly reduced migratory ability compared to that of the control group (Fig. 8). Similarly, the Transwell assay demonstrated that cell migration was inhibited when CCL14 expression was upregulated compared with that in the control group (Fig. 9).

### Relationship between CCL14 expression and tumor-infiltrating immune cells in LUAD

**Fig. 10:**
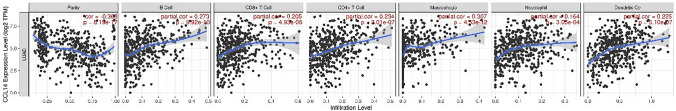
Correlation analysis between CCL14 expression and immune infiltration in patients with LUAD via TIMER

This study used the TIMER database to determine how LUAD tumor-infiltrating immune cells are correlated with CCL14 expression. Figure 10 shows the strong correlations between increased CCL14 expression and immune cell infiltration (B cells: cor = 0.273, CD8 + T cells: cor = 0.205, CD4 + T cells: cor = 0.234, macrophages: cor = 0.307, neutrophils: cor = 0.164, and dendritic cells: cor = 0.225; all *P* < 0.05).

We next used the TIMER (Table 3) and GEPIA (Table 4) databases to investigate the relationship between CCL14 expression and immune cell infiltration levels in LUAD based on immunological marker sets. Table 3 presents a detailed analysis of correlations between CCL14 expression and immune cell markers in LUAD, based on data from the TIMER database. Significant positive associations were observed with markers of various immune cell populations. For T cells, CCL14 correlated positively with CD3E (cor = 0.208, P = 3.36E−06*) and CD2 (cor = 0.153, P = 6.37E−04^*^). B cell markers showed positive correlations, with CD19 (cor = 0.297, P = 1.68E−11^*^) and CD79A (cor = 0.222, P = 6.15E−07^*^). Monocyte markers correlated positively with CD86 (cor = 0.148, P = 9.97E−04^*^) and CD115 (cor = 0.186, P = 3.11E−05^*^). Tumor associated macrophage showed significant positive correlations with CCL2 (cor = 0.095, P = 3.41E−02^*^), CD68 (cor = 0.178, p = 6.79E−05^*^), and IL10 (cor = 0.219, p = 9.38E−07^*^).

**Table 3:** Correlation analysis between CCL14 and related genes and markers of immune cells in TIMER

Description	Gene marker	LUAD			
		None		Purity	
		Core	*P*	Core	*P*
CD8 + T-cell	CD8A	0.222	3.94E−07^*^	0.070	1.18E−01
	CD8B	0.167	1.50E−04^*^	0.047	3.02E−01
T-cell (general)	CD3D	0.256	3.70E−09^*^	0.076	9.30E−02
	CD3E	0.364	1.36E−17^*^	0.208	3.36E−06^*^
	CD2	0.317	1.77E−13^*^	0.153	6.37E−04^*^
B-cell	CD19	0.421	1.51E−23^*^	0.297	1.68E−11^*^
	CD79A	0.350	2.83E−16^*^	0.222	6.15E−07^*^
Monocyte	CD86	0.286	4.55E−11^*^	0.148	9.97E−04^*^
	CD115	0.306	1.29E−12^*^	0.186	3.11E−05^*^
TAM	CCL2	0.204	3.04E−06^*^	0.095	3.41E−02^*^
	CD68	0.288	3.43E−11^*^	0.178	6.79E−05^*^
	IL10	0.340	2.09E−15^*^	0.219	9.38E−07^*^
M1 macrophage	iNOS	− 0.082	6.15E−02	− 0.032	4.73E−01
	IRF5	0.158	3.13E−04^*^	0.038	4.06E−01
	COX2	0.094	3.35E−02^*^	0.114	1.15E−02^*^
M2 macrophage	CD163	0.275	2.51E−10^*^	0.159	4.03E−04^*^
	VSIG4	0.262	1.75E−09^*^	0.168	1.87E−04^*^
	MS4A4A	0.361	1.88E−17^*^	0.251	1.50E−08^*^
Neutrophils	CD66b	0.334	6.55E−15^*^	0.344	3.55E−15^*^
	CD11b	0.267	9.24E−10^*^	0.148	9.98E−04^*^
	CCR7	0.539	0.00E+00^*^	0.427	2.68E−23^*^
Natural killer cell	KIR2DL1	0.106	1.62E−02^*^	0.058	1.98E−01
	KIR2DL3	0.072	1.04E−01	− 0.016	7.31E−01
	KIR2DL4	− 0.107	1.53E−02^*^	− 0.217	1.15E−06^*^
	KIR3DL1	0.095	3.12E−02^*^	0.027	5.48E−01
	KIR3DL2	0.048	2.79E−01	− 0.050	2.70E−01
	KIR3DL3	− 0.096	2.85E−02^*^	− 0.126	4.96E−03
	KIR2DS4	0.088	4.64E−02^*^	0.017	7.05E−01
Dendritic cell	HLA-DPB1	0.453	1.97E−27^*^	0.360	1.57E−16^*^
	HLA-DQB1	0.292	1.44E−11^*^	0.185	3.54E−05^*^
	HLA-DRA	0.389	0.00E+00^*^	0.283	1.66E−10^*^
	HLA-DPA1	0.395	0.00E+00^*^	0.301	9.27E−12^*^
	CD11c	0.296	7.46E−12^*^	0.166	2.22E−04^*^
Th1	T-bet	0.279	1.35E−10^*^	0.132	3.24E−03^*^
	STAT4	0.317	1.78E−13^*^	0.188	2.55E−05^*^
	STAT1	− 0.007	8.72E−01	− 0.139	1.96E−03^*^
	TNF-α	0.158	3.28E−04^*^	0.004	9.31E−01
Th2	GATA3	0.217	6.82E−07^*^	0.087	5.49E−02
	STAT6	0.174	7.04E−05^*^	0.196	1.12E−05^*^
Th2	STAT5A	0.362	2.02E−17^*^	0.242	5.06E−08^*^
	IL13	0.162	2.21E−04^*^	0.103	2.19E−02
Tfh	BCL6	0.135	2.24E−03^*^	0.134	2.97E−03^*^
	IL21	0.061	1.69E−01	− 0.023	6.05E−01
Th17	STAT3	0.130	3.19E−03^*^	0.146	1.14E−03^*^
	IL17A	0.043	3.31E−01	− 0.046	3.08E−01
Treg	FOXP3	0.203	3.51E−06^*^	0.042	3.52E−01
	CCR8	0.220	4.80E−07^*^	0.077	8.95E−02
	STAT5B	0.280	9.69E−11^*^	0.261	4.24E−09^*^
	TGFβ	0.322	1.01E−13^*^	0.242	5.01E−08^*^
T-cell exhaustion	PD-1	0.136	1.96E−03^*^	− 0.039	3.90E−01
	CTLA4	0.188	1.90E−05^*^	0.003	9.44E−01
	LAG3	0.048	2.81E−01	− 0.100	2.64E−02^*^
	TIM-3	0.249	1.11E−08^*^	0.102	2.30E−02^*^
	GZMB	− 0.018	6.77E−01	− 0.186	3.16E−05^*^

*CCL14* C–C motif chemokine ligand 14, *LUAD* lung adenocarcinoma, *TIMER* Tumor Immunoassay Resource

^*^P < 0.05

**Table 4:** Correlation analysis between CCL14 and related genes and markers of immune cells in GEPIA

Description	Gene Marker	LUAD			
		Tumor		Normal	
		R	*P*	R	*P*
Monocyte	CD86	0.280	2.70E−10^*^	− 0.270	0.036^*^
	CD115	0.260	4.30E−09^*^	0.014	0.920
TAM	CCL2	0.190	1.90E−05^*^	0.019	0.890
	CD68	0.340	6.70E−15^*^	− 0.069	0.600
	IL10	0.410	0.00E+00^*^	− 0.078	0.550
M2 Macrophage	CD163	0.410	0.00E+00^*^	− 0.240	0.062
	VSIG4	0.300	1.50E−11^*^	− 0.095	0.470
	MS4A4A	0.430	0.00E+00^*^	− 0.160	0.220
Neutrophil	CD66b	0.200	1.50E−05^*^	0.340	0.095
	CD11b	0.240	6.70E−08^*^	− 0.270	0.042^*^
	CCR7	0.420	0.00E+00^*^	− 0.036	0.790
T-cell (general)	CD3D	0.150	8.10E−04^*^	− 0.150	0.260
	CD3E	0.280	3.60E−10^*^	− 0.076	0.570
	CD2	0.200	7.20E−06^*^	− 0.110	0.410
B-cell	CD19	0.340	1.70E−14^*^	− 0.040	0.760
	CD79A	0.260	4.70E−09^*^	− 0.045	0.730
Dendritic cell	HLA-DPB1	0.390	0.00E+00^*^	− 0.000	1.000
	HLA-DQB1	0.150	8.80E−04^*^	− 0.029	0.830
	HLA-DRA	0.320	2.50E−13^*^	− 0.150	0.250
	HLA-DPA1	0.330	1.40E−13^*^	0.062	0.640
	CD11c	0.270	3.10E−09^*^	− 0.260	0.046^*^
Th1	T-bet	0.003	9.60E−01	− 0.220	0.100
	STAT4	0.130	4.70E−03^*^	− 0.180	0.180
	TNF-α	0.120	6.70E−03^*^	0.140	0.280
Th2	STAT6	0.100	2.30E−02^*^	0.160	0.220
	STAT5A	0.370	0.00E+00^*^	0.023	0.860
	IL13	0.110	1.50E−02^*^	0.280	0.032^*^
Tfh	BCL6	0.110	1.40E−02^*^	0.028	0.830
	IL21	0.041	3.70E−01	− 0.061	0.650
Treg	FOXP3	0.120	7.60E−03^*^	0.055	0.680
	CCR8	0.130	5.30E−03^*^	− 0.007	0.960
	STAT5B	0.270	1.10E−09^*^	0.290	0.028^*^
	TGFB1	0.170	1.80E−04^*^	0.170	0.190
T-cell exhaustion	PD-1	0.042	3.60E−01	− 0.052	0.690
	CTLA4	0.007	1.20E−01	0.040	0.760
	LAG3	− 0.038	4.10E−01	− 0.150	0.240
	TIM-3	0.270	8.40E−10^*^	− 0.290	0.026^*^
	GZMB	− 0.073	1.10E−01	− 0.280	0.030^*^

*CCL14* C–C motif chemokine ligand 14, *LUAD* lung adenocarcinoma, *GEPIA* Gene Expression Profiling Interactive Analysis

^*^P < 0.05

Table 4 presents a detailed analysis of correlations between CCL14 expression and immune cell markers in LUAD, based on data from the GEPIA database. Significant positive associations were observed with markers of various immune cell populations. For monocytes, CCL14 correlated positively with CD86 (R = 0.280, P = 2.70E−10*) and CD115 (R = 0.260, P = 4.30E−09*). Tumor-associated macrophage markers showed positive correlations, with CCL2 (R = 0.190, P = 1.90E−05*) and CD68 (R = 0.340, P = 6.70E−15*). M2 macrophage markers correlated positively with VSIG4 (R = 0.300, P = 1.50E−11*). Neutrophil markers showed significant positive correlations with CD11b (R = 0.240, P = 6.70E−08*) and CD66b (R = 0.200, P = 1.50E−05^*^). T cell markers correlated positively with CD3D (R = 0.150, P = 8.10E−04^*^), CD3E (R = 0.280, P = 3.60E−10*) and CD2 (R = 0.200, P = 7.20E−06*). B cell markers showed positive correlations with CD19 (R = 0.340, P = 1.70E−14*) and CD79A (R = 0.260, P = 4.70E−09*).

**Fig. 11:**
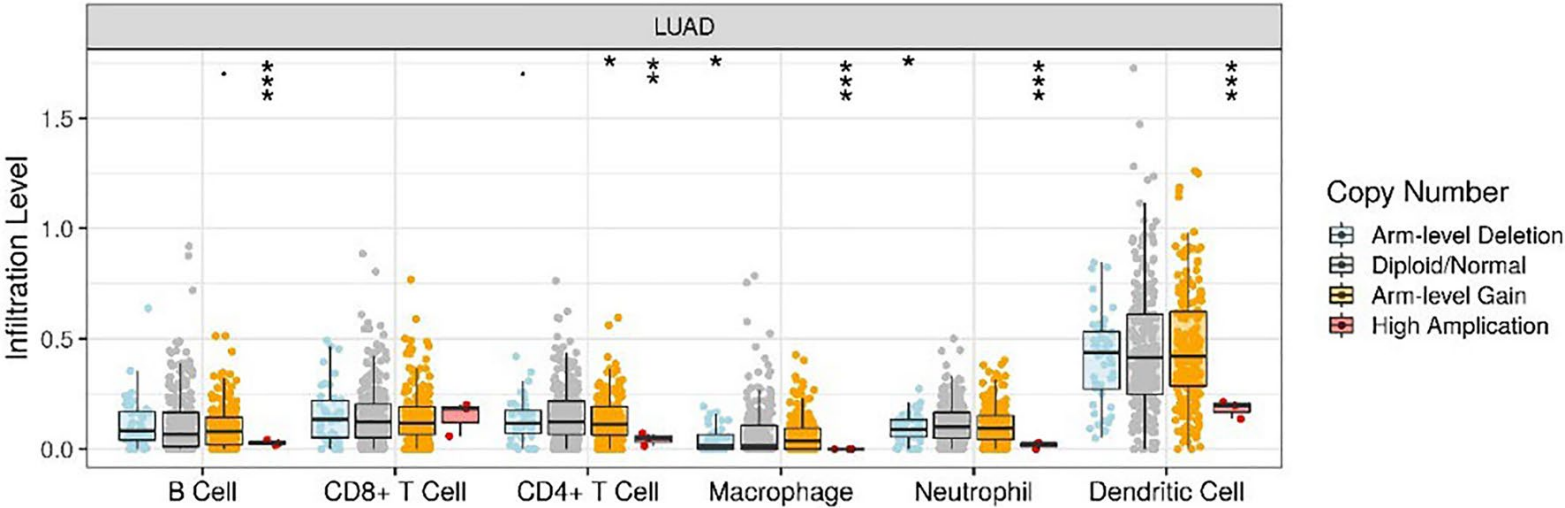
Comparison of the level of tumor invasion in LUAD and the changes in the copy number of CCL14 in different somatic cells

Additionally, we evaluated the levels of CCL14 somatic copy number alteration (SCNA) in different types of immune cells that infiltrate tumors in LUAD. Figure 11 shows that CCL14 was significantly associated with LUAD immune infiltrating cell amplification (not including CD8 + T cells), arm-level deletion, diploid/normal status, and arm-level boost.

### PPI network of CCL14 in *cancer* and enrichment analysis

**Fig. 12:**
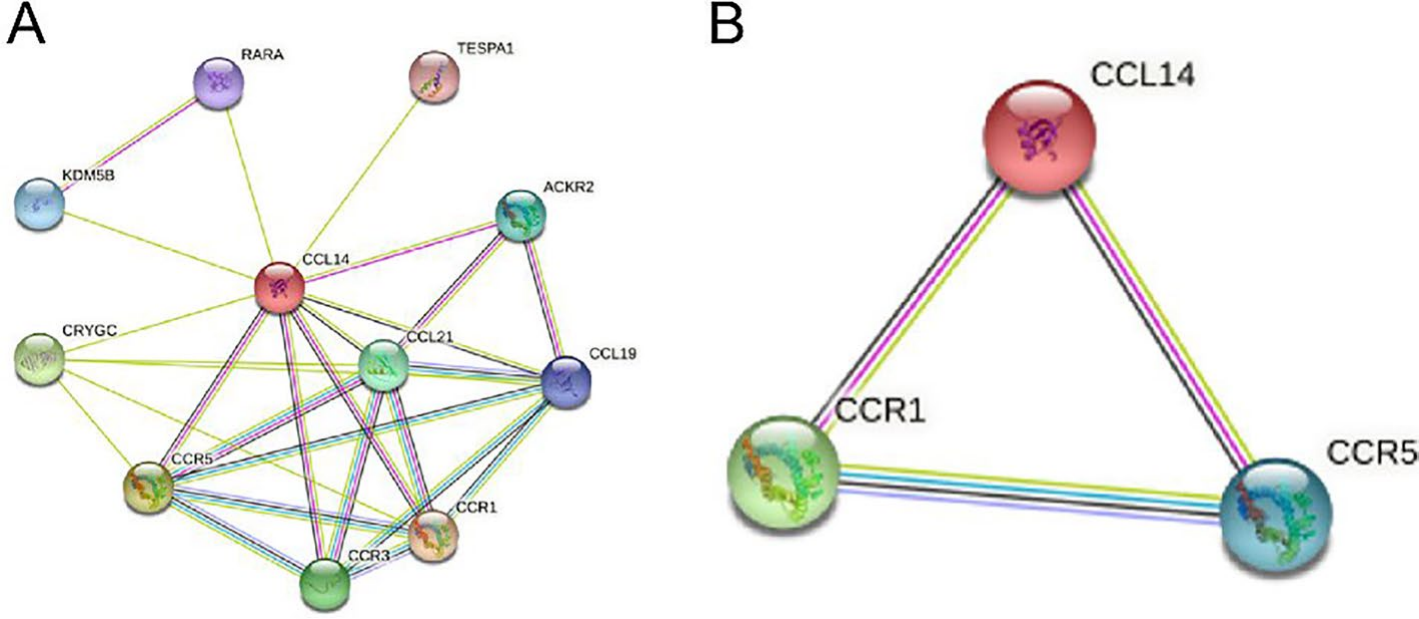
A PPI network for CCL14 was constructed in STRING (**A**) For the “medium confidence (0.400)” minimum required interaction score, ACKR2, CCL21, CCL19, RARA, CRYGC, CCR5, CCR1, KDM5B, TESPA1 and CCR3 were closely related to CCL14. **B** For the “high confidence (0.700)” minimum required interaction score, CCR1 and CCR5 were closely related to CCL14.

We performed a protein‒protein interaction (PPI) network analysis using STRING to investigate the possible connections between differentially upregulated CC chemokines. When a minimum needed interaction value of “medium confidence (0.400)” was set, the genes ACKR2, CCL21, CCL19, RARA, CRYGC, CCR5, CCR1, KDM5B, TESPA1, and CCR3 were shown to be tightly associated with CCL14 (Fig. 12A). Strong associations were observed for the expression of CCL14, CCR1, and CCR5 when a minimum interaction score of “high confidence (0.700)” was selected (Fig. 12B).

**Fig. 13:**
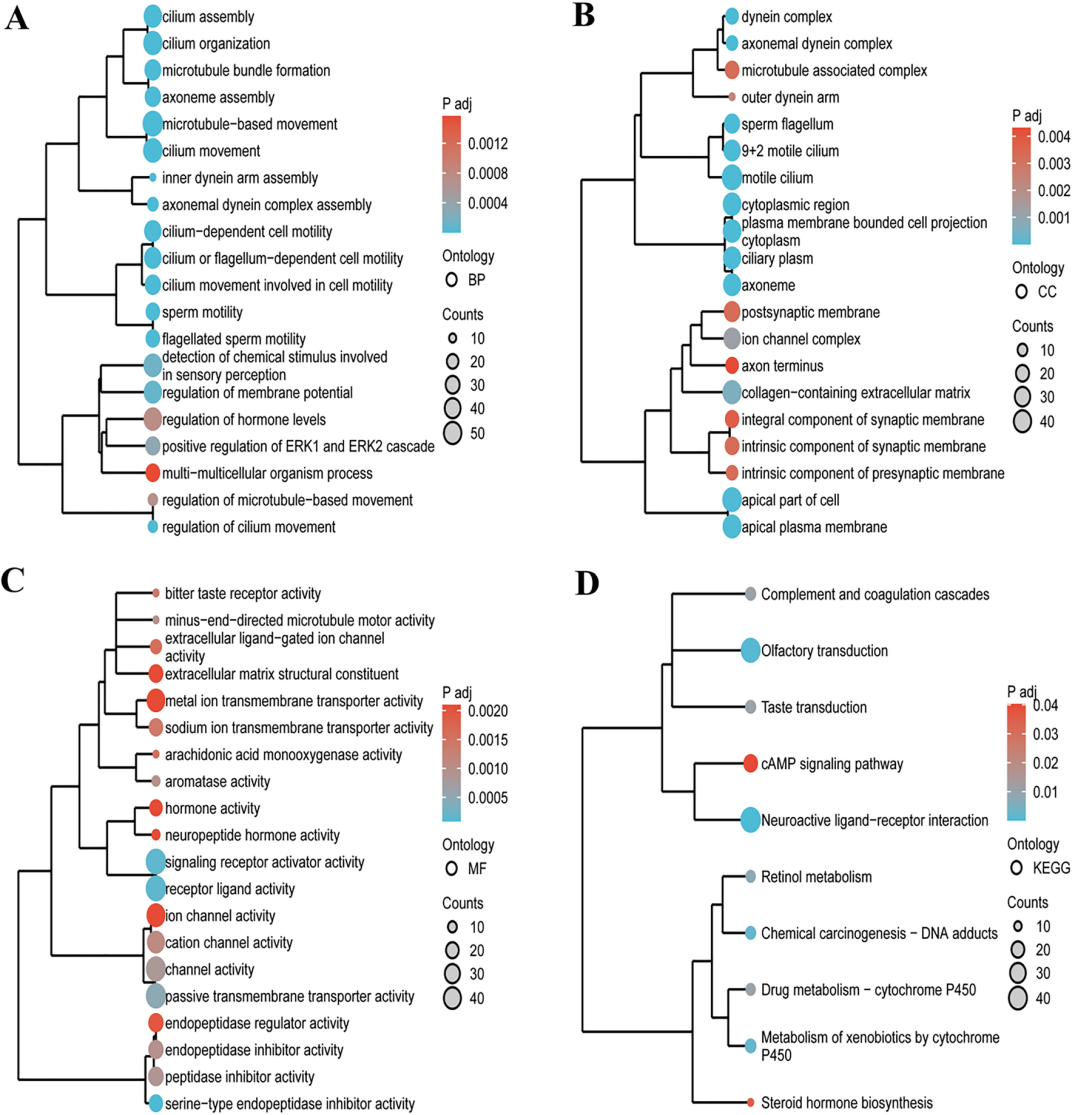
Functional enrichment analysis of differentially expressed genes associated with CCL14 expression in LUAD based on GO terms and KEGG pathways. The bubble plots show the top 20 significantly enriched terms for each category, with the size of the bubbles representing the number of genes and the color indicating the adjusted p-value. **A** KEGG pathway analysis. **B** GO Biological Process (BP) analysis. **C** GO Cellular Component (CC) analysis. **D** GO Molecular Function (MF) analysis

The GO-KEGG analysis revealed significant enrichment in various biological processes, cellular components, molecular functions, and KEGG pathways. GO analysis of biological processes showed a strong emphasis on cilium movement, axoneme assembly, microtubule bundle formation, cilium or flagellum-dependent cell motility, cilium-dependent cell motility, microtubule-based movement, cilium movement involved in cell motility, cilium organization, axonemal dynein complex assembly, and cilium assembly (Fig. 13A). Cellular component analysis identified enrichment in motile cilium, axoneme, ciliary plasm,  plasma membrane bounded cell projection cytoplasm, axonemal dynein complex, cytoplasmic region, dynein complex, and apical plasma membrane (Fig. 13B). Molecular function analysis revealed significant enrichment in diverse activities, including serine-type endopeptidase inhibitor activity, receptor ligand activity, signaling receptor activator activity, passive transmembrane transporter activity, channel activity, peptidase inhibitor activity, minus-end-directed microtubule motor activity, aromatase activity, endopeptidase inhibitor activity, and cation channel activity (Fig. 13C). The KEGG pathway analysis highlighted the importance of neuroactive ligand-receptor interaction, olfactory transduction, metabolism of xenobiotics by cytochrome P450, chemical carcinogenesis—DNA adducts, retinol metabolism, drug metabolism—cytochrome P450, complement and coagulation cascades, taste transduction, steroid hormone biosynthesis, and cAMP signaling pathway (Fig. 13D).

**Fig. 14:**
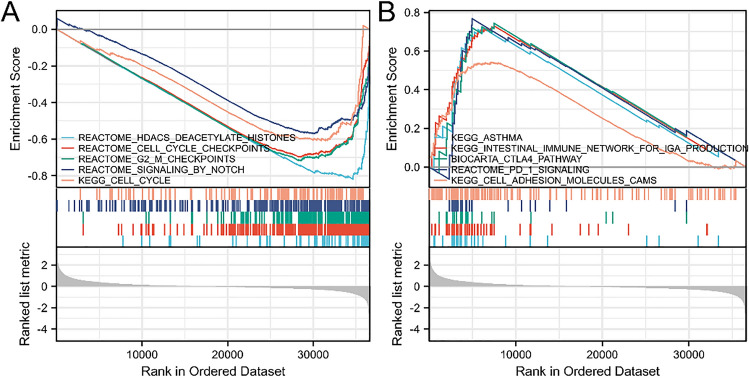
GSEA for samples with high CCL14 expression and low CCL14 expression. **A** The enriched gene sets collected from the low CCL14 expression samples. **B** The enriched gene sets in samples with high CCL14 expression

Figure 14A and B show the GSEA results, with the functional enrichment linked to low and high CCL14 expression, respectively. Table 5 shows the paths in which there were significant differences. Reduction in CCL14 expression was mostly associated with Notch signaling pathways, cell cycle checkpoints, G2/M checkpoints, and histone deacetylases (HDACs). Numerous pathways, including those involved in asthma, the CTLA4 pathway, the intestinal immunological network for IgA production, CAMs, and the programmed death 1 (PD-1) signaling pathway, have been associated with elevated levels of CCL14.

**Table 5:** Results of gene set enrichment analysis (GSEA)

Gene set name	NES	NOM *p* value	FDR
REACTOME_HDACS_DEACETYLATE_HISTONES	− 2.902	0.003^*^	0.021
REACTOME_CELL_CYCLE_CHECKPOINTS	− 2.884	0.003^*^	0.021
REACTOME_G2_M_CHECKPOINTS	− 2.782	0.003^*^	0.016
REACTOME_SIGNALING_BY_NOTCH	− 2.312	0.003^*^	0.016
KEGG_CELL_CYCLE	− 2.257	0.002^*^	0.021
KEGG_ASTHMA	2.287	0.002^*^	0.021
KEGG_INTESTINAL_IMMUNE_NETWORK_FOR_IGA-PRODUCTION	2.246	0.002^*^	0.0016
BIOCARTA_CTLA4_PATHWAY	2.181	0.001^*^	0.016
REACTOME_PD_1_SIGNALING	2.048	0.002^*^	0.016
KEGG_CELL_ADHESION_MOLECULES_CAMS	1.925	0.002^*^	0.016

*GSEA* gene set enrichment analysis, *NES* normalized enrichment score

^*^P < 0.05

## Discussion

According to the results, LUAD tissues had far lower levels of CCL14 expression than normal tissues. Sex, tumor status, and pathological stage were strongly correlated with CCL14 expression in patients with LUAD. We discovered that CCL14 is a separate prognostic factor after investigating its prognostic relevance in LUAD. The prognosis of patients with LUAD is worse when CCL14 expression is low. Compared with LUAD cell lines (NCI-H1299 and PC9), lung epithelial cells (BEAS-2B) in an artificial environment had substantially greater CCL14 mRNA expression. Compared to those in the control groups, cells whose CCL14 expression was increased exhibited less migration, invasion, and proliferation.

Based on existing research, CCL14 may influence the prognosis of LUAD patients through multiple mechanisms. CCL14 expression has been found to correlate with the infiltration levels of various immune cells and the expression of immune checkpoint genes such as PD-L1 and CTLA-4, suggesting that CCL14 may exert its effects by modulating the tumor immune microenvironment [11]. Furthermore, CCL14 might maintain the anti-tumor function of T cells by inhibiting T cell exhaustion [9, 11]. Additionally, CCL14 has been shown to have a direct inhibitory effect on tumor cells, suppressing their proliferation and promoting apoptosis [12]. At the molecular level, CCL14 negatively correlates with Notch signaling and cell adhesion molecule pathways, which are closely associated with tumor migration, indicating that CCL14 may reduce tumor metastasis by inhibiting these pathways [11]. These multifaceted actions may collectively contribute to the association between high CCL14 expression and better prognosis in LUAD patients. However, the specific roles of these mechanisms in LUAD require further functional experiments for validation.

We found that CCL14 expression was positively associated with tumor-infiltrating immune cells (TIICs) and particular immune cell genetic changes in LUAD TIICs are strongly associated with tumor progression, therapy and prognosis [13]. Chemokines impact cancer prognoses by modulating the invasion of different kinds of immune cells [8, 14]. The alterations in TIICs induced by CCL14 expression in the tumor microenvironment may influence tumor progression through multiple complex mechanisms, primarily including immune cell recruitment, immune cell activation, and tumor stroma remodeling. These mechanisms interact with each other, collectively shaping the tumor microenvironment, and consequently affecting tumor growth, invasion, and metastasis. During HIV infection, CCL14 expression has been shown to facilitate the activation of T lymphocytes, eosinophils, and monocytes [15, 16]. We performed TIMER database analysis to determine the correlations between CCL14 expression and TIICs. According to the findings of this study, the infiltration of various types of immune cells, specifically macrophages, was found to be correlated with CCL14 expression. The expression levels of macrophage markers, such as M1 markers (COX2) and M2 markers (CD163 and VSIG4), exhibited strong correlations with the expression of CCL14. In addition to upregulating the expression of CCR1 and CCR5 receptors, macrophages and T cells have been shown to induce ovarian cancer cell death and generate an antitumor immune milieu, according to prior research [17]. The CCL14/CCR1/CCR5 axis may account for the aforementioned effect, and these studies suggest that CCL14 might serve as a valuable independent marker for tumor immunity.

To examine potential associations among the CC chemokines that were differentially expressed, we subsequently conducted a PPI network analysis utilizing STRING. As predicted, interactions between CCL14, CCR1, and CCR5 expression were strongly correlated. According to reports, the CCR1/CCR5 axis is involved in the liver metastasis of colorectal cancer. This axis is responsible for recruiting various cell types and immune systems, including tumor-associated fibroblasts (CAFs), Treg-regulatory T cells, TILs, MSCs, and MDSCs, into the tumor niche [18]. In addition, our findings revealed that CCL14 was linked to functional T cells, including Tregs and fatigued T cells. GZMB, LAG3, and TIM-3 are indicators of worn-out T cells. As the primary inhibitory immunological checkpoint protein, TIM-3 has been identified and has been instrumental in the development of targeted treatments and immunotherapy [19, 20]. The majority of cancers, including lung cancer, escape the tumor immune response by inhibiting T-cell function and overexpressing inhibitory ligands, ultimately leading to tumor progression [21, 22]. Research has demonstrated that CCL14 interacts with chemokine receptors, including CCR1, CCR3, and CCR5, to control leukocyte activation and movement through Ca2 + influx [23]. Moreover, alterations in T-cell Ca2 + channels stimulate cytokine production and inhibit TIM-3 expression [24]. Therefore, through modifications to Ca^2+^ channels, CCL14 expression may control the expression of TIM-3. We hypothesize that abnormal T-cell function may be connected to the poor prognosis of patients with LUAD. In the LUAD microenvironment, CCL14 is critical for immunological escape.

To determine the functional enrichment of genes with low and high CCL14 expression, we also performed GSEA. Low CCL14 expression was mainly associated with HDACs, cell cycle checkpoints, G2/M checkpoints and Notch signaling pathways. Research has shown that histone deacetylases (HDACs) can deacetylate core histones. This deacetylation process causes chromatin compaction and the suppression of gene expression. Many genes, including those involved in cell cycle inhibition, differentiation, and apoptosis, cannot be expressed normally [25, 26]. Currently, various HDAC inhibitors are being tested in both animal and human studies. Vorinostat can increase the efficacy of paclitaxel and carboplatin in non-small cell lung cancer cells. Moreover, it has a significant inhibitory effect on NSCLC cell line growth in vitro and a much smaller effect on A549 lung cancer xenograft growth [25, 27]. Cell cycle regulation is important for the development of multicellular organisms, and cell cycle imbalance is a key process in tumorigenesis and development. The G2/M checkpoint is the typical target of cell cycle inhibition. We hypothesize that CCL14 may lead to the destruction of the G2/M checkpoint and result in the abnormal proliferation of cancer cells [28]. Based on evidence from an animal xenograft tumor model, researchers have demonstrated that elevated levels of CCL14 inhibit the growth of HCC cells, regulate cell division, and promote cell death in living creatures [12]. The Notch signaling system is essential for controlling several components in immunosuppressive settings [29]. Studies have shown that Notch signaling is involved in the regulation of lymphocytes (developing lymphocytes, B lymphocyte subsets, and helper and regulatory T cells) and the differentiation and functions of DCs [30, 31]. Among these immunologically related cells, activated CD8 + T cells are the primary actors in antitumor immune function. Furthermore, macrophages that are defective in Notch signaling have a reduced ability to deliver antigens [31, 32].

Additionally, GSEA demonstrated that increased CCL14 levels were mostly associated with pathways related to asthma, the CTLA4 pathway, the intestinal immune network for IgA formation, CAMs, and the PD-1 signaling pathway. The PD-1 immune checkpoint and CTLA-4 are crucial regulators of the T-cell response in tissues. They differ in terms of downregulation time, signaling method, and location of immunosuppression [33]. Immunotherapy based on these two checkpoint inhibitors has achieved promising results in advanced NSCLC [34]. Evidence from the latest phase 3 clinical trials showed that nivolumab plus ipilimumab combined with two cycles of chemotherapy significantly improved OS and could become a new first-line treatment option for patients with advanced NSCLC [35]. Our research also linked high levels of CCL14 expression to actions that can aggravate asthma. Evidence suggests that CCL14 prevents eosinophil recruitment in allergic airway inflammation by inducing CCR3 internalization, which in turn causes CCR3-mediated intracellular calcium release and chemotactic desensitization [36].

Our findings indicate that CCL14 may serve as a standalone predictive biomarker for LUAD and can be used to assess the extent of immune cell infiltration in tumor tissues. Future research should focus on developing strategies to enhance CCL14 expression or activity, such as CCL14 recombinant proteins or small molecules that upregulate CCL14. We propose conducting prospective studies to further validate the relationship between CCL14 expression and LUAD prognosis, and to explore the potential of combining CCL14-targeted therapies with immune checkpoint inhibitors. Additionally, evaluating serum CCL14 as a diagnostic and monitoring biomarker holds significant promise. These investigations may advance the application of CCL14 in personalized LUAD treatment, potentially improving patient outcomes.

## Conclusion

The findings of our study indicate that CCL14 plays a crucial role in determining the prognosis of patients with LUAD. Moreover, the strong correlations between CCL14 and TIICs suggest that CCL14 may have a significant influence on overall prognosis. This research was not without its limitations, however. More samples from patients with LUAD are needed to demonstrate the predictive usefulness of CCL14. Future clinical studies should verify the involvement of CCL14 in TIIC control in malignancies and its effect on the response to immunotherapy. Ultimately, CCL14 has the potential to serve as both a diagnostic tool and a therapeutic target in the context of LUAD risk assessment and management.

## Plasmid sequences

Atgaagatct ccgtggctgc cattcccttc ttcctcctca tcaccatcgc cctagggacc aagactgaat cctcctcacg gggaccttac cacccctcag agtgctgctt cacctacact acctacaaga tcccgcgtca gcggattatg gattactatg agaccaacag ccagtgctcc aagcccggaa ttgtcttcat caccaaaagg ggccattccg tctgtaccaa ccccagtgac aagtgggtcc aggactatat caaggacatg aaggagaactga.

## Data Availability

The corresponding author can provide all data used in this study upon request.
